# Pharmaceutical Pricing Policy in Greece: Toward a Different Path

**DOI:** 10.3389/fpubh.2016.00185

**Published:** 2016-08-31

**Authors:** Kyriakos Souliotis, Manto Papageorgiou, Anastasia Politi, Athanasios Athanasiadis

**Affiliations:** ^1^Department of Social and Education Policy, University of Peloponnese, Corinth, Greece; ^2^Centre for Health Services Research, Medical School, University of Athens, Athens, Greece; ^3^Department of Statistics, Athens University of Economics and Business, Athens, Greece; ^4^Foundation for Economic and Industrial Research, Athens, Greece

**Keywords:** external reference pricing, reimbursement, affordability, GDP, insurance price, Greece

## Abstract

**Background:**

Affordable, accessible, and innovation-promoting pharmaceutical care is essential to the operation of a sustainable health system. External reference pricing (ERP), a common pharmaceutical policy in Europe, suffers today from indigenous weaknesses that may cause market distortions and barriers to care, burdening mostly the weak economies, and hence, raising ethical and political worrying.

**Objectives and methods:**

A non-randomized experiment was conducted, in order to examine the influence of flexible and adaptable to health systems’ affordability ERP structures. Outcomes were assessed by measuring deviations from Greek prices’ level *ex ante*, as well as effects on pharmaceutical markets affiliated to the European ERP system.

**Results and conclusion:**

Pharmaceutical pricing models that fit prices to income and affordability are better in all aspects, as they produce fairer results, while resulting in low external costs for the European ERP network as a whole. Small sets of reference countries are preferred to large baskets, as they produce similar results, while presenting better qualities by increasing the flexibility of the reimbursement system and the transparency of the market.

## Introduction

The provision of affordable, accessible, and innovation-promoting pharmaceutical care services is an essential element of a sustainable health policy (1). In most European countries, the main purchaser of pharmaceuticals is the public sector (2), and in these quasi-monopsony conditions, national health services’ negotiating power is enhanced as to the determination of pharmaceutical prices. To this respect, the vast majority of European countries employ external reference pricing (ERP), a price-regulation method whereby a government considers the price of a medicine in a specified basket of countries in order to set its price (3). The application of ERP differs among countries, with regard to, e.g., basket size, basket composition, and re-referencing rates. Yet, a country’s ability to adjust medicinal prices to its income and affordability is limited due the method’s endogenous structural and functional characteristics.

External reference pricing is a univariate algebraic process that incorporates information solely on prices, not accounting for other key socioeconomic factors, e.g., health-care resources, health-care structure, and demographic patterns (4), in the equation. In addition, the use of extensive ERP baskets results in the increase of unmatched observations on currencies and medicinal properties (brand names, packaging, etc.), encumbering the computational process, as *ad hoc* studies illustrate (5–10). As a consequence, ERP’s sub-optimal design has a profound impact on health policies’ efficiency: it produces outcomes that present low adaptability to economies’ national income, increase market risks (e.g., arbitrage, medicine launch delays, slashed patent holders’ profitability and competitiveness, etc.), raising barriers to existing therapies and to innovation as well (2). Nevertheless, the use of large baskets of countries remains a common European practice, given that 12 European Union (EU-28) countries refer to a minimum of 15 countries (11)^1^.

Greece is a dynamic player of the European ERP system. The implementation of ERP pertains to all on-patent medicines sold in the country, directly affecting reimbursement. According to legislation, the prices of on-patent medicines are set as the average of the three lowest ex-factory prices in the EU-28. Any available price (e.g., ex-factory, wholesale, retail, hospital, insurance) is collected from official, published sources, and the necessary conversion of retail or wholesale to ex-factory price is made according to methodology and rates determined by the National Organization for Medicines (EOF) (12). Using ERP, pharmaceutical prices are reviewed (re-pricing) twice per year. Following each re-pricing, the Positive Reimbursement List is also reviewed, in order to update reimbursed prices accordingly. At the same time, Greece is included in the ERP basket of half of the EU-28 countries [14 out of 28 (3)] and is among the countries with the highest frequency of re-referencing (biannual) (2).

Since the beginning of the economic crisis in 2010, pharmaceutical expenditure has been placed in the center of fiscal consolidation and has become inextricably linked to the level of the continuously decreasing Greek gross domestic product (GDP) (13). In particular, according to the target set by the Memorandum, public pharmaceutical expenditure must not exceed 1% of GDP. For this reason, a number of cost-containment measures targeting the prices of pharmaceuticals, such as flat price reductions, the implementation of multiple rebates/discounts and ERP have been applied to obtain quick reductions in public pharmaceutical spending and thus achieve the fiscal targets. In this context, fitting flexible and unerring pharmaceutical pricing models that take into account national income and financial indicators is a matter of national necessity. The present study investigates new methodological pathways in the field of pharmaceutical pricing and reimbursement with aim to communicate additional evidence and inform health policy decision-making.

## Methodological Design

### Outline and Objectives

We examine a flexible and adaptable to the payer’s affordability ERP structure, applying a non-randomized experiment, with Greek pharmaceutical prices forming the basis of the performed comparisons. The study is based on the working hypothesis that the smaller the differential effects of interventions on Greek prices, the lower the pharmaceutical market risks, both internally and externally due to ERP’s spillover chain effects (6).

The analysis is conducted in two stages. The first stage examines the influence of small ERP baskets on the level of domestic pharmaceutical prices. The concept here is to remove procedural limitations, such as currency and price data incompatibility [as ex-factory, retail, and hospital prices can currently enter the system (14)], by using a concise, and thus, flexible ERP design. The second stage proposes the establishment of a simple model/procedure that adapts prices to the country’s affordability level. This approach is a counterproposal to the current, extraordinary, and flat pharmaceutical policy measures that are being implemented in an effort to reduce excessive spending, but disturb the normal functioning of the pharmaceutical market and health care after all (15).

### Data Selection and Arrangement

The analysis is based on a non-random experimental design, where data selection focuses on major pharmaceutical demand areas and meets certain product homogeneity requirements. The Greek Positive Reimbursement List formed the sampling frame of the selected medicines. Medicines were sorted according to their annual market share and a sample of 24% of the List’s 50 top-selling medicines in 2012^2^ (i.e., 12 out of 50) was taken. In addition, medicines were included in the analysis based on the following criteria: (1) identical properties (name, pharmaceutical form, content, packaging, and price type) among countries and (2) homogeneity of their prices’ measurement unit (ex-factory).

The selected experimental unit comprised a concise set of eight euro-zone members that practice ERP: Austria, France, Ireland, Italy, Portugal, Slovak Republic, Slovenia, and Spain. The group’s composition was assessed according to the specific financial, demographic, epidemiologic, and infrastructure criteria. Table 1 provides this information for Greece. Statistical properties for the ex-factory price variable (i.e., descriptive measures of central tendency and variation) are presented in Table 2, by group of interest (Greece and the baskets).

**Table 1:** **Socioeconomic indicators of reference countries and Greece**.

Indicator (2013)	Greece	Basket 1^a^	Basket 2^a^
GDP euro per capita (chain linked volumes) (2010)^f^	16,800^c^	24,825^d^	26,440^d^
GDP euro per capita^b^ (chain linked volumes) (2010)^f^	21,500	25,148	24,680
Total health expenditure per capita (current US$)^g^	2,146	3,229	3,374
Total health expenditure per capita^b^ (current US$)^g^	2,924	3,420	3,693
Life expectancy at birth, total (years)^h^	81.0	80.5	81.4
Life expectancy at 65, total (years)^i^	20.2	20.0	20.7
Death rate, crude (per 1,000 people)^j^	10.0	9.0	8.8
Practicing doctors (per 1,000 people)^k^	6.3	3.6	3.6
Practicing nurses (per 1,000 people)^k^	3.6	7.6	7.8^e^

*^a^Mean value*.

*^b^Data on 2009*.

*^c^Provisional*.

*^d^Provisional for Spain*.

*^e^Data on Austria include nurses employed in hospital*.

*^f^European Commission. Eurostat. Available from: http://ec.europa.eu/eurostat/tgm/table.do?tab=table&init=1&plugin=1&pcode=tsdec100&language=en. Accessed 16 January 2016*.

*^g^The World Bank. Available from: http://data.worldbank.org/indicator/SH.XPD.PCAP. Accessed 10 January 2016*.

*^h^The World Bank. Available from: http://data.worldbank.org/indicator/SP.DYN.LE00.IN. Accessed 10 January 2016*.

*^i^European Commission. Eurostat. Available from: http://appsso.eurostat.ec.europa.eu/nui/submitViewTableAction.do*.

*^j^The World Bank. Available from: http://data.worldbank.org/indicator/SP.DYN.CDRT.IN. Accessed 10 January 2016*.

*^k^OECD (16)*.

**Table 2:** **Basic descriptive statistics of the performance characteristic (ex-factory price)**.

	Scenario 1	Scenario 2
**Greece**		
Sum (€)^a^	1,589.27	1,979.75
Median (€)	36.82	41.15
Arithmetic mean (€)	198.66	164.98
SD (€)	307.38	257.72
Minimum (€)	9.62	9.62
Maximum (€)	735.91	735.91
Range (€)	726.29	726.29
Coefficient of variation	1.54	1.56
**Basket**		
Sum (€)	13,646.26	10,713.89
Median (€)	42.85	42.47
Arithmetic mean (€)	213.22	178.56
SD (€)	316.30	267.05
Minimum (€)	8.10	8.10
Maximum (€)	965.15	938.03
Range (€)	957.05	929.93
Coefficient of variation	1.48	1.50

*^a^Equal to parameter “*G*” (Eq. 8)*.

Total observations (i.e., 72) are arranged by country and medicine, according to the [*P_N_*_ ×_*_ m_*] block matrix cited in formula (Eq. 5). To achieve experiment repeatability, two data arrangement scenarios were conducted, forming two experimental units, respectively. Each scenario thus pertained to one experimental unit and Greece, which was set as the comparison base. The experimental unit of Scenario 1 included the original basket of eight referenced countries. Scenario 2 reduced the basket to a subset of five countries (France, Ireland, Italy, Portugal, and Spain). Both scenarios involved equal number of observations, as presented in formula (Eq. 4).

Between-countries comparisons of price distributions are performed using the statistical test of paired samples *t*-test of the SPSS software. The hypotheses of equality of mean ex-factory price between countries, as well as between a country and the basket mean are tested. The level of statistical significance is set at 0.05.

(1) $$n=\{\begin{array}{cc} 8, & \text{Scenario }1\\ 5, & \text{Scenario }2 \end{array}$$

(2) $$N=\{\begin{array}{cc} 9, & \text{Scenario }1\\ 6, & \text{Scenario }2 \end{array}$$

(3) $$m=\{\begin{array}{cc} 8, & \text{Scenario }1\\ 12, & \text{Scenario }2 \end{array}$$

(4) $$N\times m=72=\{\begin{array}{cc} 9\times 8, & \text{Scenario }1\\ 6\times 12, & \text{Scenario }2 \end{array}$$

(5) $${\left[P\right]}_{N\times m}=\{\begin{array}{c} {\left[\begin{array}{c} g_{i}\\ r_{i,j} \end{array}\right]}_{9\times 8}\\ {\left[\begin{array}{c} g_{i}\\ r_{i,j} \end{array}\right]}_{6\times 12} \end{array}=\{\begin{array}{c} \left[\begin{array}{c} \begin{array}{ccc} g_{1} & \cdots & g_{8} \end{array}\\ \begin{array}{ccc} r_{1,1} & \cdots & r_{1,8}\\ \vdots & \ddots & \vdots \\ r_{8,1} & \cdots & r_{8,8} \end{array} \end{array}\right],\text{Scenario }1\\ \left[\begin{array}{c} \begin{array}{ccc} g_{1} & \cdots & g_{12} \end{array}\\ \begin{array}{ccc} r_{1,1} & \cdots & r_{1,12}\\ \vdots & \ddots & \vdots \\ r_{5,1} & \cdots & r_{5,12} \end{array} \end{array}\right],\text{Scenario }2 \end{array}$$

where *n*: the number of reference countries included in the basket; *N*: total number of countries, including Greece; *m*: the number of medicines; *g_i_*: the Greek price of the *i*th medicine (*i* = 1, 2, … , *m*); *r_i_*_,_*_j_*: referenced price: the price of the *i*th medicine in the *j*th reference country (*i* = 1, 2, … , *m*; *j* = 1, 2, … , *n*); [*P_N_*_ ×_*_ m_*]: the general block matrix formula of Greek and referenced prices; ${\left[\begin{array}{c} g_{i}\\ r_{i,j} \end{array}\right]}_{9\times 8}$: the block matrix of Greek and referenced prices in Scenario 1; and ${\left[\begin{array}{c} g_{i}\\ r_{i,j} \end{array}\right]}_{6\times 12}$: the block matrix of Greek and referenced prices in Scenario 2.

### The Concise Basket Proposition

Differential effects of using concise baskets of reference countries on Greece’s prices are measured with the performance indicator CBI (Eq. 6). CBI yields the difference in percentage points, between two quantities: the Greek sum of prices “*G*” and the expected sum of prices by basket, “*E*,” which is defined as the basket’s sum of pharmaceutical prices divided by the number of countries included in the basket (Eqs 7 and 8).

(6) $$\text{CBI}=\left[\left(\frac{E}{G}-1\right)\times 100\right]\%$$

(7) $$\begin{array}{cc} E=\frac{1}{n}\sum_{j=1}^{n}\sum_{i=1}^{m}r_{i,j}=\{\begin{array}{c} \frac{1}{8}\sum_{j=1}^{8}\sum_{i=1}^{8}r_{i,j},\text{Scenario }1\\ \frac{1}{5}\sum_{j=1}^{5}\sum_{i=1}^{12}r_{i,j},\text{Scenario }2 \end{array} & \begin{array}{l} i=1,2,\ldots ,m\\ j=1,2,\ldots ,n \end{array} \end{array}$$

(8) $$\begin{array}{cc} G=\sum_{i=1}^{m}g_{i}=\{\begin{array}{c} \sum_{i=1}^{8}g_{i},\text{Scenario }1\\ \sum_{i=1}^{12}g_{i},\text{Scenario }2 \end{array} & i=1,2,\ldots ,m \end{array}$$

where *G*: Greek sum of prices; *E*: expected sum of prices of a country included in the basket of Greece; and CBI: indicator of concise-basket effects.

### Application of Insurance Price in Pharmaceutical Reimbursement

We define as insurance price (“IP”) of a medicine the pharmaceutical reimbursement price that is adjustable to the country’s affordability level. Thus, IP is calculated as a function of two variables: the first variable is the expected sum of prices of a reference country included in the basket of Greece, i.e., the parameter “*E*,” as previously presented (Eq. 7) and the second is a weight that reflects Greece’s financial status. The financial status weight, denoted as “*a*,” relies on the concept of “affordability index” presented in the literature, which correlates countries’ mean GDP per capita to the corresponding European average (2). In particular, “*a*” is equal to Greece’s GDP per capita (GDP_G_) divided by the basket’s mean GDP per capita $\left({\bar{\text{GDP}}}_{\text{B}}\right)$ (Eqs 10–12), with “*a* < 1” indicating that Greek income is lower compared to the average income of the basket. IP equals the product of “*a*” and “*E*,” as shown in Eq. 9.

During the decade 2004–2013, Greece lost a fifth of its income (−19.6%, in per capita terms) (17). Non-stationarity of GDP per capita, however, renders the variable “*a*” time-dependent. In addition, official and final data on GDP_G_ are published with considerable delay, as according to Eurostat, the most recent finalized data on GDP_G_ are traced back in 2010 (17).^3^ To overcome these weaknesses which can bias both the process and the results, 2009 data have been used in the analysis, as these data are final and also refer to a period before the economic crisis (17),^4^ enabling the drawing of more objective conclusions.

All involved GDP per capita indicators in the construction of variable “*a*” refer to 2009. The indicator of insurance price effects (IPI) yields the difference between the value of the insurance price “IP” and the Greek sum of prices “*G*,” in percentage points (Eq. 13).

(9) IP=a×E

(10) a=a1a2

(11) $$a_{1}={\text{GDP}}_{\text{G}}$$

(12) $$a_{2}={\bar{\text{GDP}}}_{\text{B}}$$

(13) $$\text{IPI}=\left[\left(\frac{\text{IP}}{G}-1\right)\times 100\right]\%$$

where IP: insurance price; *a*: affordability weight; IPI: indicator of insurance price effects; GDP_G_: Greece’s GDP per capita; and ${\bar{\text{GDP}}}_{\text{B}}$: arithmetic mean of the GDP per capita of the basket.

### Estimation of External Costs

External reference pricing models in essence constitute functions that calculate arithmetic means. Almost half of EU-28 (13/28) use the average price of their entire ERP basket, while others calculate the average price of a selected part of the basket (e.g., of a small subset of the lower prices, or the lowest value itself, etc.). Eight out of the 11 countries that refer to Greece in their ERP basket apply the first method (3). The impact (external costs) from Greece’s ERP process transformation (i.e., basket shrinking and “IP” application) on the affiliated European pharmaceutical markets was estimated by applying the average price of the whole basket (most common ERP method). Indicative values were produced using the pharmaceutical prices of this experiment. Three indices are presented in Eqs 14–16: “ERPC,” “EC_1_,” and “EC_2_.” ERPC measures the outcome of a typical ERP process for a randomly selected European country “C” that includes Greece in its basket; EC_1_ is an index that measures external costs resulting from the shrinking of Greece’s basket, and EC_2_ is an index that measures respective costs resulting from the application of Greece’s IP procedure. Detailed results are provided in Table 4.

(14) $$\text{ERPC}=\frac{P}{b}=\frac{\sum_{i=1}^{b}p_{i}}{b}=\frac{p_{1}+p_{2}+\ldots +p_{b}}{b}$$

(15) $${\text{EC}}_{1}=\left[\left(\frac{P+\text{CBI}\times p_{\text{G}}}{P}-1\right)\times 100\right]\%=\left(100\times \text{CBI}\times \frac{p_{\text{G}}}{P}\right)\%$$

(16) $${\text{EC}}_{2}=\left[\left(\frac{P+\text{IPI}\times p_{\text{G}}}{P}-1\right)\times 100\right]\%=\left(\text{IPI}\times \frac{p_{\text{G}}}{P}\right)100\%$$

where ERPC: the outcome of a typical ERP process exercised by a random country “C,” which includes Greece in a basket of size “*b*”; *p_i_*: the medicinal price in the *i* referenced country included in C’s basket, *i* = 1, 2, … , *b*; *b*: the number of countries included in C’s basket; *p*_G_: the *p_i_* value when the referenced country is Greece; *P*: the sum of all *p_i_*’s, in the basket of size “*b*,” *i* = 1, 2, … , *b*; EC_1_: the index that measures external costs (in C) due to Greece’s basket shrinking; and EC_1_: the index that measures external costs (in C) due to Greece’s IP application.

## Results

The next paragraphs provide point estimates of the differential basket and reimbursement revision effects on Greece’s prices. Quantitative results for the potential impact of the proposed model on the country’s external environment are additionally presented. The microeconomic, macroeconomic, and demographic indicators that compose the research conditions under which the present experiment was conducted are also discussed.

The formed baskets represent countries with similar demographic trends, as indicated by the data on life expectancy and death rates presented in Table 1. However, homogeneity between Greece and its baskets is reduced when health resources are taken into account. In particular, medical staff density in the population varies significantly between Greece and the baskets. Yet, this variation is independent from the baskets’ composition, considering Greece’s ranking above the OECD average with regards to this particular health-care parameter (16). The picture is completely reversed when the geographical dispersion of the nursing personnel is examined, with the number of practicing nurses per 1,000 people less than half in Greece compared to the baskets (Table 1). Another difference between Greece and the baskets concerns total per capita health expenditure. Greece’s per capita health expenditure is below the corresponding average of the baskets. The latter is consistent with the rich theoretic background on the positive relation between GDP and public health expenditures per capita in OECD (18) and the fact that Greece’s income was lower in comparison with the baskets.

Income correlations constitute fundamental characteristics of this experiment where differential IP effects hinge on. The baskets represented countries of a higher income, on average, compared to Greece (Table 1). Basket 1 is placed below the European average (€25,500 and €28,300 in EU-28 and the Euro-area, respectively) (17), while basket 2 exceeds the EU-28 average. The application of IP reduces the level of current home prices by 6.49–7.47% (Scenarios 1 and 2, respectively) (Table 3), causing though no severe impact abroad, considering the small effects that vary per product and overall between −1.71% (medicine “M_6_,” Scenario 2) and −0.64% (medicine “M_10_,” Scenario 1), as Table 4 shows.

**Table 3:** **Point-estimates of parameters and variables**.

Index	Description	Scenario 1	Scenario 2
*G*	Sum of prices, Greece (€)	1,589.27	1,979.75
*E*	Expected sum of prices, referenced country (€)	1,705.78	2,142.78
IP	Insurance price (€)	1,486.08	1,831.86
CBI	Indicator of concise-basket effects (%)	+7.33	+8.24
IPI	Indicator of insurance price effects (%)	−6.49	−7.47
*a*	Affordability weight (%)	87.12	85.49

**Table 4:** **Values of external-cost indices**.

Medicinal product			EC_1_		EC_2_	

ID	Ex-factory price (€)		*S*_11_	*S*_12_	*S*_21_	*S*_22_

	Min	Max	%	%	%	%
M_1_	12.93	36.43	na	1.63	na	−1.47
M_2_	27.00	42.55	0.83	1.47	−0.74	−1.33
M_3_	39.78	52.87	0.74	1.29	−0.66	−1.17
M_4_	473.61	938.03	0.84	1.62	−0.74	−1.47
M_5_	35.70	42.60	na	1.73	na	−1.57
M_6_	33.56	68.11	0.91	1.88	−0.80	−1.71
M_7_	33.07	42.38	0.82	1.48	−0.73	−1.34
M_8_	273.88	386.40	na	1.32	na	−1.20
M_9_	32.96	81.70	0.75	1.22	−0.66	−1.11
M_10_	8.10	17.11	0.73	1.33	−0.65	−1.20
M_11_	34.97	117.39	na	1.21	na	−1.10
M_12_	635.40	770.00	0.89	1.56	−0.79	−1.41
All	8.10	938.03	0.85	1.52	−0.76	−1.38

*S_11_: EC_1_ – Scenario 1; S_12_: EC_1_ – Scenario 2; S_21_: EC_2_ – Scenario 1; S_2_: EC_2_ – Scenario 2; na: not applicable*.

Regarding the performance characteristic, Greek prices were lower, compared to the mean price of the baskets, yet they presented higher variability, as indicated by the comparative central tendency and variation measures presented in Table 1. There were no statistical differences among the price distributions of the Scenario 1 (eight countries), except for the pair “France-Slovak Republic,” where French prices were lower (*p*-value = 0.05). In the smaller country set of the Scenario 2, Ireland appeared as an outlier, having statistically higher prices compared to France, Greece and Portugal (*p*-values 0.017, 0.022, and 0.024, respectively). This scenario yielded statistically lower Greek prices by 7.61% (*p*-value = 0.05) compared to the mean of the basket. CBI effects are in accordance with these statistical results, retaining however, their small range (<1%) across scenarios, considering that basket downsizing to five to eight countries caused Greek prices’ increase by 8.24% at the most (Table 3).

## Discussion

External reference pricing, a common policy tool used worldwide to control prices of pharmaceuticals, is – in its present form – no longer considered sufficient in terms of operational efficiency and adaptability to payers’ available income, and has raised a lot of discussion and concerns among policy-makers, academia, and the industry. In this context, the present analysis produces new evidence regarding changes that could be incorporated in the ERP tool, to restore its efficacy.

A non-randomized experiment was conducted in order to examine the impact from the implementation of an ERP model that is flexible and adaptable to health systems’ affordability level. This experiment used Greek pharmaceutical prices as a key node of the European ERP network and tested the impact of ERP basket reconstruction and price-model fitting based on fiscal parameters. Results are examined with regard to the impact of differential price effects on both the Greek and other EU pharmaceutical markets, assessing the proposed model in terms of its theoretic value and contribution.

Regarding the insurance price proposition, the model yielded small price effects in the two examined scenarios, despite the identified statistical differences between the involved price populations. Small price reductions though not only are consistent with the budgetary targets of the country but also deter collateral financial damages of restrictive pharmaceutical policies, such as public revenue losses, which is currently a serious fiscal issue in Greece (15).

Moreover, with its small price decreases, the proposed IP model poses a disincentive for parallel exports of pharmaceuticals, which in the past has been an intense phenomenon (19), restricting thus the role of arbitragers and thereby reducing the risk of pharmaceutical market shortages and barriers to pharmaceutical care (20). Considering barriers to medical care a topical health-care issue in Europe (16), this model property is very important from a general health policy perspective, and utilizable in Greece, where unmet health needs currently affect 9% of the population on average, while specific patient populations encounter even higher rates (e.g., 13% for multiple sclerosis patients) of barriers to medication (21).

In this context, the application of a bivariate pricing model that takes into account not only prices but also countries’ affordability level adjusts prices in the best interest of the public sector and the beneficiaries. Beyond these encouraging results, the model’s moderate effects also restrict unwanted price effects and potential damage in other countries to low percentages, i.e., around 1% (in absolute terms).

In support of these findings, which are consistent with other published studies (2), the estimated values of external cost indices (EI_1_ and EI_2_) are in fact overestimations. The mean size of EU baskets that include Greece equals 21 (11),^5^ whereas this work examined considerably smaller basket sizes (≤8 countries), which decrease the denominator of the quantity "pGP", thus increasing the value of indices, “EC_1_” and “EC_2_” (Eqs 15 and 16).

In terms of the question of theoretic contribution, the presented model proposed the use of smaller baskets in ERP practicing. Taking into account the complex system of Greece with multiple sources of data, we conclude that structures that are more flexible bypass bureaucracy, enable computational transparency, and increase homogeneity of referenced countries. Furthermore, as the moderate increasing effects (<10%) of the basket-downsizing model on the Greek prices indicate, this improvement is achieved without causing serious price and market volatilities.

## Conclusion

Technological progress, which shifts quality and prices of pharmaceutical products upwards (18, 22, 23), as well as the rapid change in the demographic and epidemiological profile of the population (24), create a dynamic grid of long-term challenges for health-care systems, which are facing major pressures on their budgets. In light of the above, health-care decision makers must not stay inert. Advancing the quantity and quality of information incorporated in pharmaceutical pricing methodology, as suggested by the present analysis, is a road that is worth being paved for all: the economy, the health-care system, and the health status of the population.

## Author Contributions

All authors contributed equally to the conduct of the study and the development of this manuscript.

## Conflict of Interest Statement

The authors declare that the research was conducted in the absence of any commercial or financial relationships that could be construed as a potential conflict of interest.

## Footnotes

^1^Based on calculations of the authors

^2^Based on records of Greece’s E-Government of Social Security (“IDIKA”)

^3^Based on calculations of the authors

^4^Based on calculations of the authors

^5^Based on calculations of the authors
